# Formation of Degenerate Band Gaps in Layered Systems

**DOI:** 10.3390/ma5061055

**Published:** 2012-06-07

**Authors:** Anton I. Ignatov, Alexander M. Merzlikin, Miguel Levy, Alexey P. Vinogradov

**Affiliations:** 1 Institute for Theoretical and Applied Electromagnetics, Russian Academy of Sciences, Izhorskaya Street 13, Moscow 125412, Russia; E-Mails: a.m.merzlikin@gmail.com (A.M.M.); a-vinogr@yandex.ru (A.P.V.); 2 Department of Physics, Michigan Technological University, 1400 Townsend Drive, Houghton, MI 49931-1295, USA; E-Mail: mlevy@mtu.edu

**Keywords:** anisotropic photonic crystal, magneto-photonic crystal, band gap, frozen mode, Borrmann effect, optical Tamm state

## Abstract

In the review, peculiarities of spectra of one-dimensional photonic crystals made of anisotropic and/or magnetooptic materials are considered. The attention is focused on band gaps of a special type—the so called degenerate band gaps which are degenerate with respect to polarization. Mechanisms of formation and properties of these band gaps are analyzed. Peculiarities of spectra of photonic crystals that arise due to the linkage between band gaps are discussed. Particularly, it is shown that formation of a frozen mode is caused by linkage between Brillouin and degenerate band gaps. Also, existence of the optical Borrmann effect at the boundaries of degenerate band gaps and optical Tamm states at the frequencies of degenerate band gaps are analyzed.

## 1. Introduction

In the last two-three decades the electromagnetics of inhomogeneous media has developed very rapidly. A great advance in theoretical electromagnetics was stimulated by the transfer (the so-called “mapping”) of wave phenomena from the quantum solid state realm. The results of this mapping are the development of photonic crystals [1,2,3], diffusion of light [4], weak localization (coherent backscattering) and Anderson localization of light [4], optical Tamm states [5,6,7,8,9,10,11,12,13,14,15,16] and so on.

However, in spite of the similarity of wave phenomena in various areas of physics, there is a fundamental difference between electromagnetics and quantum mechanics: the main object of quantum mechanics (neglecting effects connected to spin), the wave function, is a scalar quantity, whereas electric and magnetic fields are vectors.

In some particular cases this difference is of no importance. For example, the problem of a vector wave traveling through a one-dimensional system of isotropic layers can be reduced to that of a scalar wave identical to a quantum mechanical problem with a proper change of notation [4,15]. But if a wave scatters on two- or three-dimensional objects or layers made of anisotropic materials, it becomes important to take in account the vector nature of the electromagnetic wave.

In this article we show that the vector nature of electromagnetic waves results in a number of peculiarities of wave transport. For example, one-dimensional photonic crystals (PCs) made of anisotropic and magneto-optical materials exhibit band gaps of a special type as a result of hybridization of Bloch waves of different polarizations [6,17,18,19,20,21,22,23,24,25,26,27,28]. 

We will confine ourselves to the case of one-dimensional (1D) PCs. It is known that the formation of band gaps (BGs) is due to Bragg reflection from the PC elementary cells [29]. In other words BGs originate from the constructive interference of partial waves, reflected back from boundaries of different elementary cells. The condition for the Bragg reflection corresponding to BGs is

(1) $$\text{Re}k_{Bl}\Lambda =\pi m$$

where $k_{Bl}$ is the Bloch wave number, Λ is the PC period, m is an integer. There is a simple qualitative interpretation of this condition in the case of a low contrast PC. In such a PC the index of refraction (and therefore the local wave number $k\left(\vec{r}\right)$) varies little through the PC elementary cell, and $k_{Bl}\approx k\left(\vec{r}\right)$ [29]. Thus condition (1) is the condition for the optical path of a partial wave passing an elementary cell back and forth by a multiple of 2π. We refer to such band gaps as Brillouin BG because it may form only at the Brillouin zone boundary. 

The band structure of PCs composed of gyrotropic or anisotropic materials has peculiar features not present in PCs made of isotropic materials. 1D PCs composed of layers of uniaxial crystals with angularly offset optical axes (lying in the plane of the layers) for adjacent layers were shown [17,18,19,20,30] to have special BGs in their spectra, not at the boundary of Brillouin zone, but inside. Similar BGs were shown [6,21,22,23,24,25,26,27,28] to appear for photonic crystals with layers made of gyrotropic materials and anisotropic uniaxial materials with collinear optical axes for all layers.

## 2. Formation of Degenerate Band Gaps

### 2.1. Formation of Degenerate Band Gaps Inside a Passing Band

**Figure 1:**
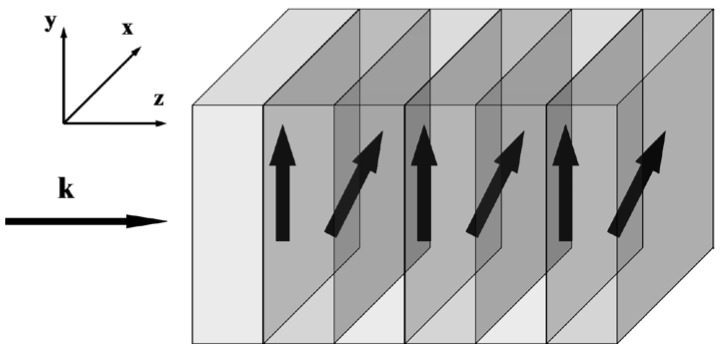
One-dimensional stratified PC with layers made of uniaxial materials. Thick arrows indicate orientations of the optical axes of the layers (in the plane of the layers). The left arrow k is the wave vector of an incident wave.

Let us consider the formation of BGs in a PC having elementary cells composed of two layers made of uniaxial materials. The optical axes of the layers are assumed to lie in the plane of layers (see Figure 1).

**Figure 2:**
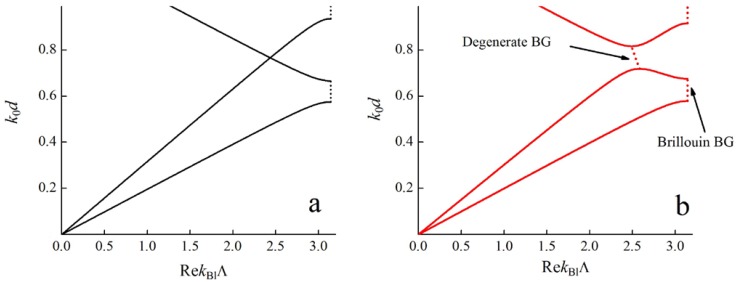
Dispersion curves of a PC with two uniaxial layers of the same thickness d per unit cell. (Λ=2d) Parameters of the first layer: ordinary dielectric permittivity $\varepsilon_{1}^{ord}=2.0$, extraordinary one $\varepsilon_{1}^{ext}=5.0$. Parameters of the second layer: ordinary dielectric permittivity $\varepsilon_{2}^{ord}=3.0$, extraordinary one $\varepsilon_{1}^{ext}=8.0$. Solid lines correspond to the frequencies of the pass bands, dashed lines correspond to the frequencies of band gaps. (**a**) The optical axes of all the layers are collinear; (**b**) The optical axes of adjacent layers are misaligned by an angle α=0.5 rad.

First let us assume that the optical axes of all the materials are collinear. In that case the Bloch waves have well-defined polarizations (either ordinary or extraordinary), and the Maxwell equations may be divided into two independent parts and each Bloch wave is indifferent to the anisotropy of the PC materials. In Figure 2(a) the Bloch dispersion curves are shown for that case.

However, in the case of misaligned optical axes, an ordinary wave can partly turn into an extraordinary wave (and *vice versa*) when reflecting from layer boundaries. Thus, the eigen-solutions become hybridized, dispersion curves interconnect and a BG appears (see Figure 2b) at frequencies around the intersection point of the dispersion curves for ordinary and extraordinary Bloch waves [17]. This BG (as is clear from the Figure 2b) forms for the two dispersion branches simultaneously; in other words, its formation is degenerate with respect to polarization. That is why we call this kind of BG degenerate (DBGs) [27]. In general DBGs may appear not at the boundary but inside the Brillouin zone. In this connection it should be noted that this type of BG is also called “intra-Brillouin”. However in the next section it will be shown that BGs of this kind may form not only inside the Brillouin zone, but also at the intersection of Brillouin BGs at the boundary of the Brillouin zone.

At the frequencies corresponding to the DBGs, the real and imaginary parts of the Bloch wave numbers are the same sign for both dispersion curves [22]. In contrast, Brillouin BGs (which form at the boundary of the Brillouin zone according to (1)) appear at different frequencies in anisotropic PCs for different dispersion branches.

Degenerate BGs can also form [27] for PCs with layers made of gyrotropic materials and anisotropic uniaxial materials even if the optical axes of all layers are collinear. However, in this case, the hybridization of ordinary and extraordinary Bloch waves is due to the Faraday effect on the polarization of the waves in the gyrotropic layers. It should be pointed out that hybridization of different polarizations and formation of BGs similar to the aforementioned degenerate BGs may also occur in many other structures such as two- and three-dimensional PCs [19,21] and periodic waveguides [31,32,33].

Degenerate BGs (DBGs) happen at the so-called exchange Bragg condition [17]

(2) (RekBl1+RekBl2)Λ=2πm

where $k_{Bl1}$ and $k_{Bl2}$ are the Bloch wave numbers corresponding to different dispersion branches. Condition (2) has a simple qualitative explanation in the case of a PC with material parameters varying little in space and with weak hybridization between ordinary and extraordinary polarizations. In such a PC the refraction coefficients for ordinary and extraordinary components (and therefore the local wave numbers $k^{ord}\left(\vec{r}\right)$ and $k^{ext}\left(\vec{r}\right)$) vary little through a PC elementary cell. The ordinary polarization component scatters weakly into the extraordinary one (and *vice versa*), $k_{Bl1}\approx k^{ord}\left(\vec{r}\right)$ and $k_{Bl2}\approx k^{ext}\left(\vec{r}\right)$. When passing an elementary cell in the forward direction, a phase incursion of a partial wave corresponds to an ordinary refraction coefficient, while in the backward direction it corresponds to an extraordinary refraction coefficient in a PC layers. Thus, an additional condition (2) arises for the total phase incursion of a partial wave passing an elementary cell back and forth by a multiple of 2π.

**Figure 3:**
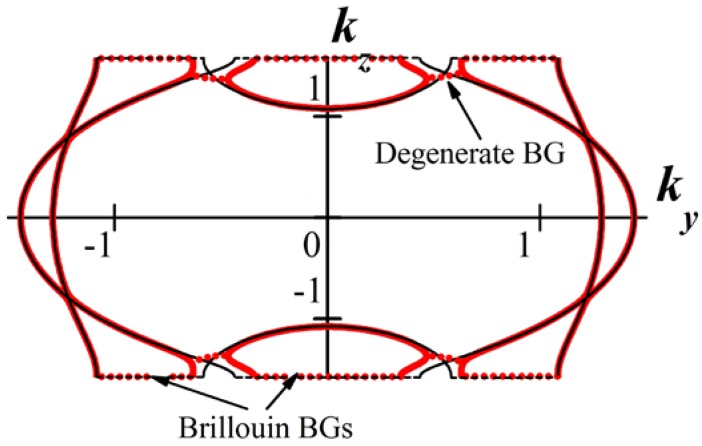
Isofrequency curves for magnetophotonic crystal with two layers of the same thickness d in a period, Λ=2d. $k_{0}d=0.9$ is a wave number in vacuum. The parameters for the first, anisotropic, layer of the period are: ordinary dielectric permittivity $\varepsilon_{1}^{ord}=8.0$, extraordinary one $\varepsilon_{1}^{ext}=2.0$. The parameters for the second, gyrotropic, layer of the period are: diagonal dielectric permittivity $\varepsilon_{2}=3.0$, non-diagonal components of dielectric tensor for the case of non-zero external magnetic field ±*ig* = ±0.3*i*. Black curves correspond to the case of zero external magnetic field, red curves correspond to non-zero external magnetic field. The two black curves correspond to s- and p-polarizations. Solid curves correspond to propagating waves, dotted curves correspond to band gaps. The optical axes of anisotropic layers are parallel to the plane of incidence (Oyz).

For simplification throughout the paper we fix the angle of incidence to the normal incidence case and represent the formation of DBG in terms of dispersion curves. However, one may fix the frequency and consider the formation of degenerate BGs by varying the angle of incidence. Let us consider, in brief, degenerate BGs for the case of oblique incidence. In Figure 3 isofrequency curves (*i.e.*, dependences of normal Bloch wave vector component $k_{z}$ for different polarizations on tangential wave vector component $k_{y}$ at a fixed frequency) for a layered magnetophotonic crystal are depicted. Black curves correspond to the case of zero external magnetic field, red curves correspond to a non-zero external magnetic field. The gyrotropy of magnetic layers induced by an external magnetic field leads to coupling of s- and p-polarized waves. As a result, degenerate BGs form near some of the intersections of isofrequency curves for s- and p-polarizations.

### 2.2. Formation of Degenerate Band Gaps Inside a Brillouin Band Gap

It should be noted that DBGs can form not only outside Brillouin BGs, but also inside as well. This becomes possible when Brillouin BGs corresponding to different dispersion branches intersect.

**Figure 4:**
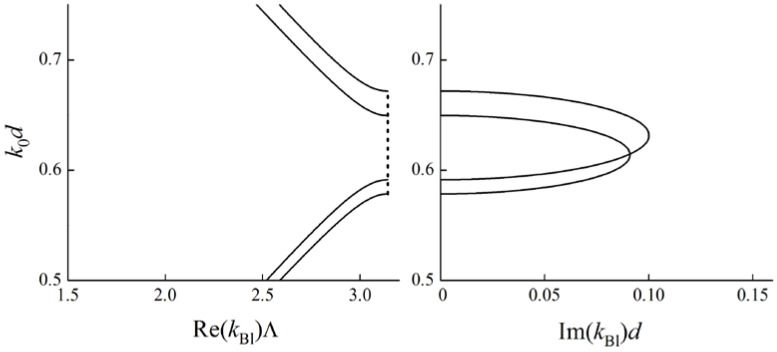
Dispersion curves are calculated for a PC with two layers of the same thickness d in a period, Λ=2d. $k_{0}$ is a wave number in vacuum. The parameters for the first layer of the period are: ordinary dielectric permittivity $\varepsilon_{1}^{ord}=5.0$, extraordinary one $\varepsilon_{1}^{ext}=7.8$. The parameters for the second layer of the period are: ordinary dielectric permittivity $\varepsilon_{2}^{ord}=7.5$, extraordinary one $\varepsilon_{2}^{ext}=5.4$. In the left figure for Re(kBl)Λ solid lines correspond to pass bands, dashed lines correspond to band gaps.

Indeed, let us re-examine the case of an anisotropic PC considered above (Figure 1) with new parameters of the layers. Assume the optical axes in all the layers to be collinear and the Brillouin BGs corresponding to ordinary and extraordinary polarizations to intersect. For this case the dispersion curves (frequency dependence of real and imaginary parts of the Bloch wave numbers) are depicted in Figure 4. Ordinary and extraordinary polarizations are not hybridized in the Bloch waves and therefore a DBG does not appear.

**Figure 5:**
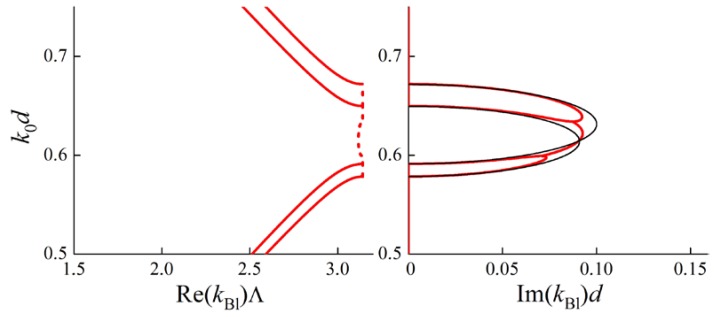
Bloch dispersion curves (red lines) for a PC with the parameters as in Figure 4, but with misaligned optical axes of adjacent layers. A DBG appears at the intersection of the Brillouin BGs. At the frequencies of the DBG both real and imaginary parts of the Bloch wave numbers for different dispersion curves are equal. Black lines being the same as in Figure 4 are shown for reference. The angle between optical axes of the adjacent layers is 0.08 rad. In the left figure for Re(kBl)Λ solid lines correspond to pass bands, dashed lines correspond to band gaps.

In the case of misaligned optical axes in adjacent layers, ordinary and extraordinary polarizations become hybridized in the Bloch waves. A DBG arises at frequencies near the intersections of the black lines (for imaginary parts of $k_{Bl}$) (see Figure 5). The curves in Figure 5 are calculated for a PC with the same material parameters of the layers as for the Figure 4. The only difference is the misalignment of optical axes of adjacent layers: the angle between them is 0.08 rad. Figure 5 shows that a DBG forms at the intersection of Brillouin BGs. At the frequencies of the DBG imaginary parts of Bloch wave numbers for both the branches are equal (up to a sign). The real parts are also equal (up to a sign), but do not lie at the boundary of the Brillouin zone, but inside.

Thus, the fact that the real part of a Bloch wave number lies inside the Brillouin zone is the indicator of a DBG.

**Figure 6:**
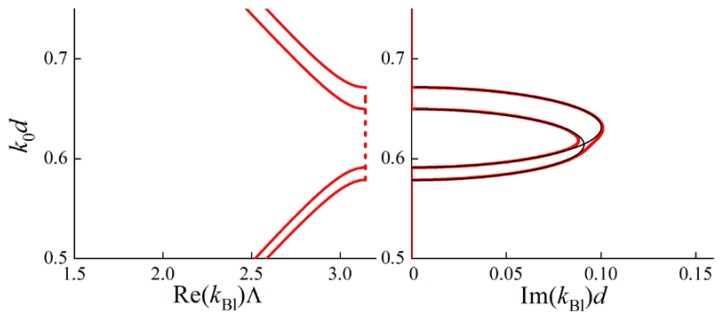
Bloch dispersion curves (red lines) for a PC with two layers of the same thickness d in a period. The optical axes of adjacent layers are misaligned by an angle of 0.3 rad. The parameters for the first layer of the period are: ordinary dielectric permittivity $\varepsilon_{1}^{ord}=5.0$, extraordinary one $\varepsilon_{1}^{ext}=5.4$. The parameters for the second layer of the period are: ordinary dielectric permittivity $\varepsilon_{2}^{ord}=7.5$, extraordinary one $\varepsilon_{2}^{ext}=7.8$. A DBG does not appear at the intersection of Brillouin BGs. The figure shows that the intersection of dispersion curves for different dispersion branches is not the only condition for a DBG to appear. Black lines corresponding to the case of collinear optical axes (the same as in Figure 4) are shown for reference. In the left figure for Re(kBl)Λ solid lines correspond to pass bands, dashed lines correspond to band gaps.

However, the conditions for a DBG to appear at the Brillouin BG frequencies are more complicated than just the intersection of dispersion curves for different dispersion branches. As an illustration let us consider another PC with two layers of the same thickness d in a period. Obviously, in the case of collinear optical axes in all the layers, the dispersion curves are the same as in Figure 4. But the misalignment of the optical axes (in the plane of layers) does not lead to the formation of a DBG (see Figure 6).

## 3. Properties of Degenerate Band Gaps

One of the main reasons for PCs to be actively studied is the existence of band gaps in their spectra. This feature allows PCs to be used as filters, dielectric mirrors, reflective walls of resonators and waveguides [34,35]. Near the boundaries of Brillouin BGs such effects as near-zero group velocity of the Bloch waves [36] and the Borrmann effect [37,38,39,40,41,42] also manifest themselves. This last consists in a spatial redistribution of the energy of a Bloch wave in a PC primitive cell: at the top and bottom frequencies of a Brillouin BG most of the electromagnetic energy of a wave concentrates in different locations inside a primitive cell, either in materials of high or low permittivity. For more details see the corresponding subsection or the references [37,38,39,40,41,42].

In this section we discuss the aforementioned effects in connection with degenerate BGs and analyze the peculiarities of spectra of PCs that arise due to the linkage between Brillouin and degenerate BGs or due to the linkage between degenerate BGs of various origins.

### 3.1. Linkage Between Brillouin and Degenerate Band Gaps. Formation of the So-Called “Degenerate Band Edge” and Frozen Mode

In the previous section we have shown that DBGs may form at the intersection of Brillouin BGs in anisotropic of gyrotropic PCs (Figure 5). In this section we present a more detailed study of the peculiarities of a PC’s band structure arising from the proximity between Brillouin and degenerate band gaps. 

**Figure 7:**
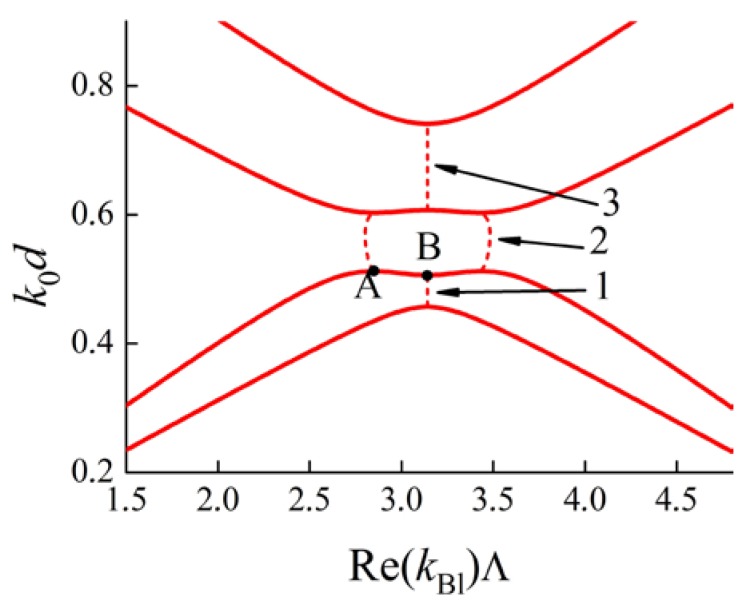
Frequency dependence $k_{0}d$ on Re(kBl)Λ for a multilayer PC (d—thicknesses of all layers, Λ=2d is a PC period). The solid lines correspond to propagating waves, the dashed lines correspond to BGs. **1** and **3** are Brillouin BGs, **2** is a DBG. **А** is a DBG boundary, **B** is a Brillouin BG boundary.

Consider the dispersion curves of a PC with a Brillouin and a degenerate BG located close to each other. The dispersion curves Re(kBl(k0)) for the PC are depicted in Figure 7. There are three frequency ranges 1, 2, 3 in the figure (dashed lines), where Im(kBl(k0))≠0, thus constituting band gaps. Based on the values of Re(kBl(k0)) we can identify ranges 1 and 3 as Brillouin BGs and range 2 as a DBG. The lower boundary of the DBG is marked with a letter A and the upper boundary of the Brillouin BG 1 is marked with a letter B.

It is known that in the vicinity of a boundary of a Brillouin BG the frequency dependence on a Bloch wave number is described by a second-order parabola [36]. The same behavior for the dispersion curves occurs in the neighborhood of a DBG boundary [36]. Therefore the group velocity of a Bloch wave approaches zero as the frequency approaches the boundary of a BG.

**Figure 8:**
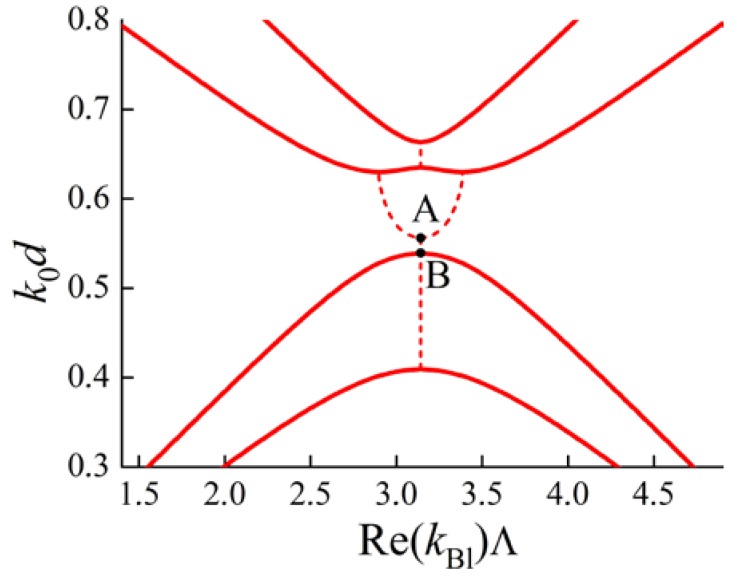
Frequency dependence $k_{0}d$ **on** Re(kBl)Λ for a multilayer PC ( d—thicknesses of all layers, Λ=2d is a PC period). The solid lines correspond to propagating waves, the dashed lines correspond to BGs.

So, near point B (Figure 7) the $k_{0}\left(k_{Bl}\right)$ dependence (for propagating waves) can be approximated by a parabola

(3) $$k_{0}\left(k_{Bl}\right)=a+b{\left(k_{Bl}-\frac{\pi }{\Lambda }\right)}^{2}+c{\left(k_{Bl}-\frac{\pi }{\Lambda }\right)}^{4}$$

where b>0. Indeed, as is clear from Figure 7, the dispersion curve is convex downwards. After a slight variation of parameters of the PC the locations of points A and B are also slightly changed. Point A steadily moves along the solid curves to the left or right and may even move up (across the point B) along the Brillouin zone boundary $k_{Bl}=\frac{\pi }{\Lambda }$ (see Figure 8).

In this case, the dependence $k_{0}\left(k_{Bl}\right)$ near point B may be approximated by the Parabola (3) with b<0.

**Figure 9:**
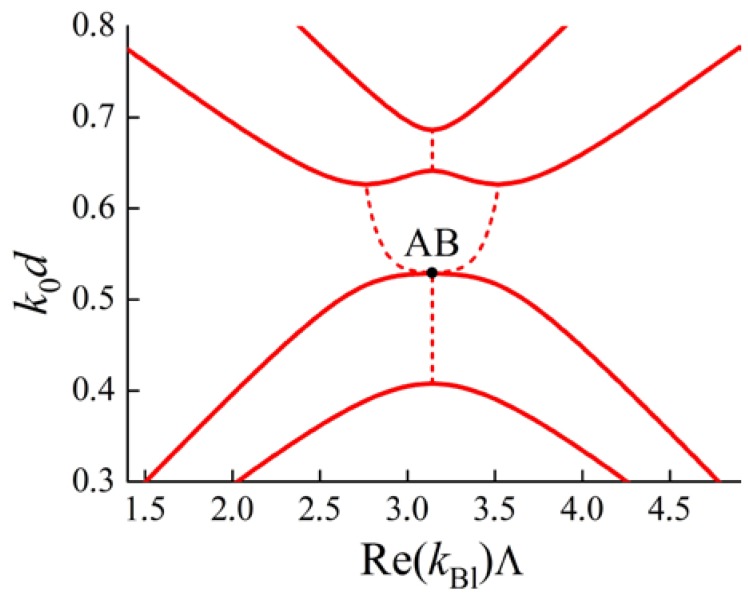
Frequency dependence $k_{0}d$ on Re(kBl)Λ for a multilayer PC (d—thicknesses of all layers, Λ=2d is a PC period). The solid lines correspond to propagating waves, the dashed lines correspond to BGs.

As long as B is a regular boundary of a BG then b≠0. But b>0 when point A is to the left of B, and b<0 when point A is above B. The dispersion curves of the PC modify continuously from that in the Figure 7 to that in the Figure 8. Therefore the contact point of A and B must be the condition for b=0 (see Figure 9). The dispersion dependence near point B is described by a fourth-order parabola.

(4) $$k_{0}\left(k_{Bl}\right)=a+c{\left(k_{Bl}-\frac{\pi }{\Lambda }\right)}^{4}$$

**Figure 10:**
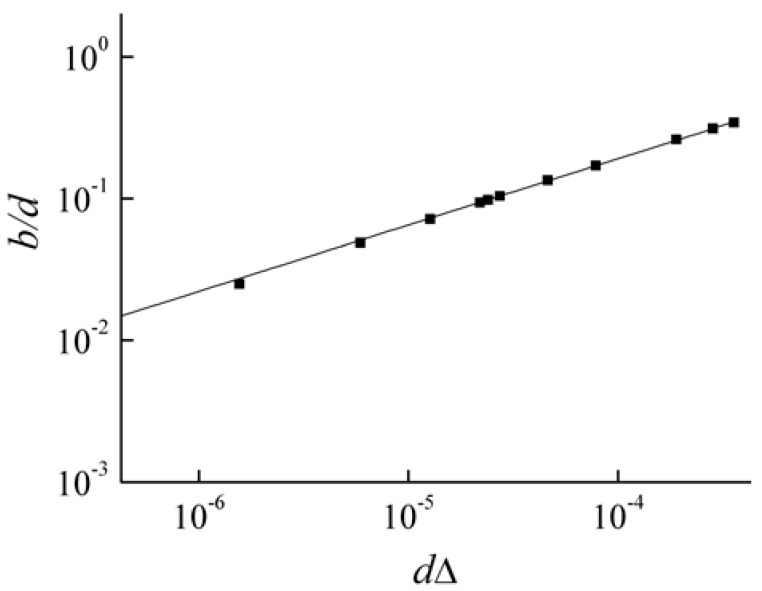
The dependence of the coefficient b in (3) on the distance Δ (along the frequency axis) between the boundaries A and B of degenerate and Brillouin BGs. The parameters for the PCs are cited in the text. The squares are the result of numerical simulations, the solid line is a trend line.

Below, we present results of numerical simulations that confirm the above arguments. Figure 10 shows the dependence of b on the distance Δ between points A and B (along the frequency axis). Simulations were carried out for a number of PCs with a period corresponding to two layers of the same thickness d made of anisotropic uniaxial materials. The optic axes of all the layers were taken to lie in the plane of layers. The angle between the optic axes of adjacent layers is 0.5 rad. Ordinary dielectric permittivities of the first and the second layers are $\varepsilon_{1}^{ord}=5.0$ and $\varepsilon_{2}^{ord}=6.5$, respectively. Extraordinary dielectric permittivities of the first and the second layers varied from 6.2 to 6.53 and from 5.7 to 6.03 respectively. We can see in Figure 10 the coefficient b tending to zero as the distance Δ tends to zero.

All numerical simulations in this subsection were carried out for the case of anisotropic PCs. However the same results occur in the case of magnetophotonic crystals that also can exhibit degenerate BGs.

So we have found a point of contact for the boundaries of degenerate and Brillouin BGs to be the so-called degenerate band edge [36], *i.e.*, the boundary $k_{0}^{*}$ of a BG with the dispersion curve behaving as a fourth-order parabola $k_{0}-k_{0}^{*}\propto {\left(k_{Bl}-k_{Bl}^{*}\right)}^{4}$ in the vicinity of $k_{0}^{*}$. Both degenerate BGs and degenerate band edges are specific features of PCs made of anisotropic and gyrotropic materials and not possible for PCs made of isotropic non-magnetic materials (this statement is true only in the case of 1D PCs).

At the frequency of a degenerate band edge the Bloch waves travel with zero group velocity (which is also true at frequencies corresponding to the regular edges of BGs). But degenerate band edges are interesting due to the possibility that the Bloch waves may also travel with near-zero dispersion in group velocity. Thus a wave packet at the frequencies near the frequency of a degenerate band edge would have vanishing group velocity and not be blurred. Such modes with zero group velocity and its dispersion are called frozen modes [43,44]. Recently frozen modes in the spectra of PCs and other structures have been actively studied in connection with their possible application to non-linear optics, telecommunications and optical computing [45,46,47]. On the topic of slow and frozen light occurring and the merging of band gaps for waves in waveguides see also [48,49,50,51].

### 3.2. Linkage Between Anisotropic and Gyrotropic Degenerate Band Gaps

In the previous section we mentioned two different mechanisms of hybridization of polarization in Bloch waves that lead to the formation of degenerate BGs. The first one (we refer to the corresponding DBGs as anisotropic DBGs) follows from different orientations of anisotropy axes in different layers of a PC, the second one (we refer to the corresponding DBGs as gyrotropic DBGs) follows from Faraday rotation of the polarization in gyrotropic layers and works even if the orientations of anisotropy axes in all the layers are the same. Anisotropic and gyrotropic DBGs form around the same frequency of intersection of two dispersion curves (see the intersection of curves in Figure 2a). The width of a DBG depends on the degree of hybridization of ordinary and extraordinary polarizations in a Bloch wave, but the central frequency of a DBG remains the same and independent of the mechanism of hybridization. Therefore the question arises: what characteristics does a DBG have if the two mechanisms of polarization hybridization work simultaneously, namely if some PC components are gyrotropic and the optical axes in different layers are misaligned? In particular, what is the width of such a combined anisotropic-gyrotropic DBG, and can one mechanism of BG formation compensate the other mechanism so that a combined BG closes up?

In the present subsection we study the characteristics of degenerate BGs in 1D stratified PCs with all the layers generally made up of anisotropic uniaxial materials with magnetically-induced gyrotropy. Some of the results presented in this subsection have been published in [52]. The optical axes in adjacent layers are in general misaligned (all lying in the plane of the layers). The characteristics of the DBGs are calculated by use of T-matrices [6,17,53,54] and analytically by use of perturbation theory [27,29] in the approximation of small periodic alterations of media parameters (low contrast PCs). The method of small perturbations used illustrates well enough the concept of polarization hybridization that we discussed only qualitatively before.

Waves are supposed to propagate only normally to the layers. The axis normal to the layers is denoted as z (see the coordinate system in Figure 1).The external stationary magnetic field inducing gyrotropy is directed along z and the optic axes of all materials lie in the plane of the layers. Therefore the permittivity tensor (as a function of z) of the structure may be written in principal axes as:

$$\hat{\varepsilon }\prime (z)=\left(\begin{array}{ccc} \varepsilon_{x}(z) & -ig(z) & 0\\ ig(z) & \varepsilon_{y}(z) & 0\\ 0 & 0 & \varepsilon_{y}(z) \end{array}\right)$$

where $\varepsilon_{x}(z)$, $\varepsilon_{y}(z)$, g(z) are periodic real functions of z. The difference between $\varepsilon_{x}(z)$ and $\varepsilon_{y}(z)$ is caused by natural anisotropy, whereas g(z) describes the magnetically-induced gyrotropy. However the optical axis related to natural anisotropy may change their orientation, rotating about the z axis by an angle α(z), also depending periodically on z. That is why, in the coordinate system xyz, (see Figure 1) common to the whole PC, the permittivity tensor is $\hat{\varepsilon }(z)=\hat{S}(z)\hat{\varepsilon }\prime (z){\left(\hat{S}(z)\right)}^{-1}$, where $\hat{S}(z)$ is the rotation matrix about the z axis by the angle α(z), which is a periodic function of z:

$$\hat{S}(z)=\left(\begin{array}{ccc} \cos\alpha (z) & -\sin\alpha (z) & 0\\ \sin\alpha (z) & \cos\alpha (z) & 0\\ 0 & 0 & 1 \end{array}\right)$$

Therefore

(5) $$\hat{\varepsilon }(z)=\left(\begin{array}{ccc} \varepsilon_{x}(z)\cos^{2}\alpha (z)+\varepsilon_{y}(z)\sin^{2}\alpha (z) & \frac{\sin2\alpha (z)}{2}(\varepsilon_{x}(z)-\varepsilon_{y}(z))-i\varepsilon (z) & 0\\ \frac{\sin2\alpha (z)}{2}(\varepsilon_{x}(z)-\varepsilon_{y}(z))+i\varepsilon (z) & \varepsilon_{y}(z)\cos^{2}\alpha (z)+\varepsilon_{x}(z)\sin^{2}\alpha (z) & 0\\ 0 & 0 & \varepsilon_{y}(z) \end{array}\right)$$

From the structure of tensor $\hat{\varepsilon }(z)$, and from the assumption that the waves propagate normally to the layers, we conclude that the waves are transverse. Thus we arrive at the following Maxwell equation for the electric field:

(6) $$\frac{d^{2}E}{dz^{2}}+k_{0}^{2}\hat{\varepsilon }(z)E=0$$

where tensor $\hat{\varepsilon }(z)$ is determined by Equation (5).

We solve Equation (6) using perturbation theory with the assumption that the periodic alterations $\Delta \varepsilon_{x}(z)$, $\Delta \varepsilon_{y}(z)$, Δg(z) in the functions $\varepsilon_{x}(z)$, $\varepsilon_{y}(z)$, ε(z), g(z) are small compared to their average values, independent on z, and that α(z) is small compared to π. Perturbation theory gives (see Appendix for details) the width $\Delta k_{0}^{\text{GA}}$ of a DBG in the presence of the two mechanisms of hybridization. In the case of a PC with two layers in a unit cell, the formula for $\Delta k_{0}^{\text{GA}}$ is quite simple:

(7) $$\Delta k_{0}^{\text{GA}}={\left[{\left(\Delta k_{0}^{\text{A}}\right)}^{2}+{\left(\Delta k_{0}^{\text{G}}\right)}^{2}\right]}^{\text{1/2}}$$

where $\Delta k_{0}^{\text{A}}$ and $\Delta k_{0}^{\text{G}}$ are the widths of the DBG in the presence of only anisotropic or only gyrotropic mechanisms, respectively. In terms of the components of tensor (5) the anisotropic mechanism of hybridization corresponds to α(z)≠const and g(z)≡0, the gyrotropic mechanism corresponds to α(z)=const and g(z)≠0.

So, in the case of a bilayer PC, the width of the combined DBG is uniquely determined by using the “Pythagorean theorem” only as a function of the widths of BGs, each related to only one formation mechanism [52]. Below we present the results of exact calculations using the T-matrix method [6,17] that confirm the correctness of Equation (7).

**Figure 11:**
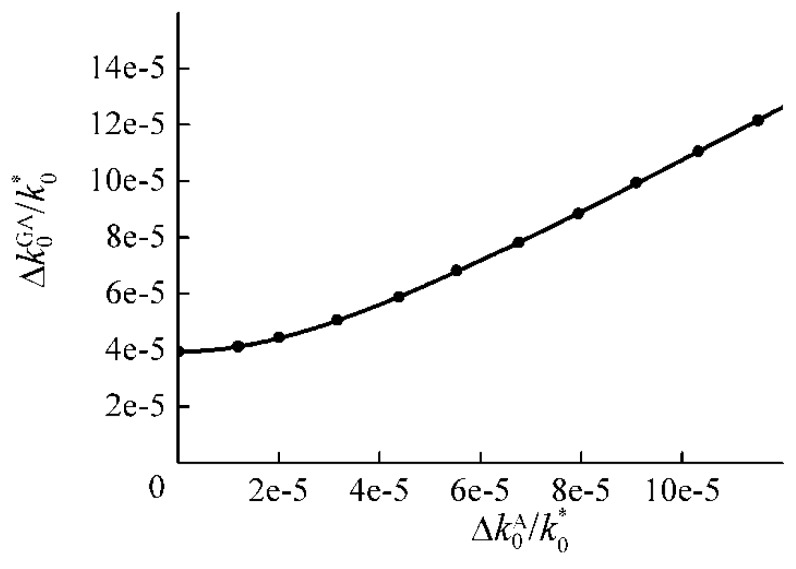
The dependence of $\Delta k_{0}^{GA}$ on $\Delta k_{0}^{A}$ at a fixed $\Delta k_{0}^{G}$ for a bilayer PC. The solid curve is calculated using the “Pythagorean theorem”, and points are calculated numerically by using the transfer matrix method. The parameters for the first layer of the PC are: $\varepsilon_{x}^{first}=5.8$, $\varepsilon_{y}^{first}=5.6$, $g^{first}=2\times 10^{-3}$; the parameters for the second layer are: $\varepsilon_{x}^{second}=8.43$, $\varepsilon_{y}^{second}=6.84$, $g^{second}=0$. The thicknesses of both the layers are the same. $k_{0}^{*}$ is the central frequency of the BGs.

In Figure 11 the dependence of $\Delta k_{0}^{GA}$ on $\Delta k_{0}^{A}$ at a fixed $\Delta k_{0}^{G}$ is shown. All the BG widths are normalized to the frequency $k_{0}^{*}$ of the center of BGs. Here the values of the material parameters of the PC were taken to be close to that of real materials. The solid curve is described by Equation (7), whereas the points present the result of an exact calculation of $\Delta k_{0}^{GA}$ using the T-matrix method. $\Delta k_{0}^{G}$ and $\Delta k_{0}^{A}$ were also calculated using the T-matrix method. We can see very good agreement between the results given by Equation (7) and by the T-matrix method. The widths of the BGs are rather small because of weak gyrotropy and anisotropy.

It should be pointed out that Equation (7) is still valid even if we take considerably larger values (by an order of magnitude) for the anisotropy and the gyrotropy in the PC materials. In practice, such an increase may be realized in composite structures. Particularly micro-resonator structures can be utilized to enhance the effective anisotropy and/or effective gyrotropy [6,8].

**Figure 12:**
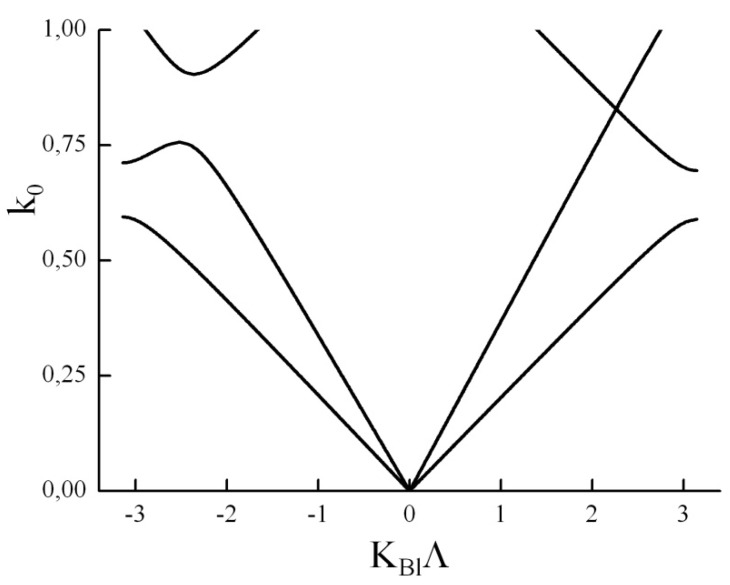
Dispersion curves of a PC with four layers in a unit cell. A DBG exists for RekBl<0 whereas at RekBl>0 a DBG is closed.

If there are three or more layers in a PC unit cell a linkage between DBGs of different types (anisotropic and gyrotropic) could be more complicated (see Appendix). In particular, one mechanism of hybridization can compensate the other one so that the DBG closes up. In this case the dispersion curves are non-symmetric relative to $k_{Bl}=0$ (see Figure 12). Thus a PC with three or more layers in a period may exhibit non-reciprocal properties. According to perturbation theory, the “Pythagorean theorem” (7) is valid as soon as dispersion curves are symmetrical in the presence of the two hybridization mechanisms, no matter how complex a unit cell is. In [55] it was shown that a 1D PC may have non-symmetric (relative to $k_{Bl}=0$) dispersion curves only if its period consists of three or more layers. Thus (7) is always valid for a low contrast bilayer PC.

### 3.3. Absence of the Optical Borrmann Effect at the Boundary of a Degenerate Band Gap

Almost all applications of PCs are related to the existence of band gaps, which allow one to design high-Q microcavities and optical waveguides [35]. Another group of PC applications is based on the possibility of controlling the field distribution in a primitive cell by varying the incidence angle and/or the light frequency. The effect of a field-redistribution inside a PC unit cell depending on optical frequency is called optical Borrmann effect [37,38,39,40,41,42]. Under certain conditions the variation of the frequency can shift the nodes and antinodes of the electromagnetic field distribution to desired locations inside the primitive cell. For example, such locations can be in the middle of magneto-optical layers of a magnetophotonic crystal or in the middle of the layer displaying strongest absorption. This makes it possible to enhance the desired linear or non-linear optical response of one of the constituents of the photonic crystal, while suppressing undesirable effects. In particular, one can enhance the Faraday or Kerr effects in magnetophotonic crystals and/or decrease losses in the same PC [40,41,42].

The optical Borrmann effect is more pronounced at the edges of BGs [35,37,38,39,40,41,42,56]. The nature of the effect may be easily elucidated for the particular case of the Borrmann effect for a Brillouin BG of an isotropic 1D PC. At the lower and upper boundaries of a Brillouin BG Bloch wave numbers are the same and equal to $k_{Bl}\left(k_{0}^{-}\right)=k_{Bl}\left(k_{0}^{+}\right)=\pi /\Lambda$. The Bloch modes $E\left(z,k_{0}^{-}\right)$ and $E\left(z,k_{0}^{+}\right)$ at the lower and upper boundaries of the BG must be orthogonal to each other [35].

(8) $$\int_{0}^{\Lambda }E\left(z,k_{0}^{-}\right)E^{*}\left(z,k_{0}^{+}\right)dz=0$$

Because Maxwell equations are Hermitian for the lossless 1D case. Here z is a coordinate normal to the PC layers, $k_{0}^{-}$ and $k_{0}^{+}$ are frequencies of the lower and the upper edges of the Brillouin BG. Thus, $E\left(z,k_{0}^{-}\right)$ has to spatially concentrate in the areas where $E\left(z,k_{0}^{+}\right)$ tends to zero and *vice versa*.

**Figure 13:**
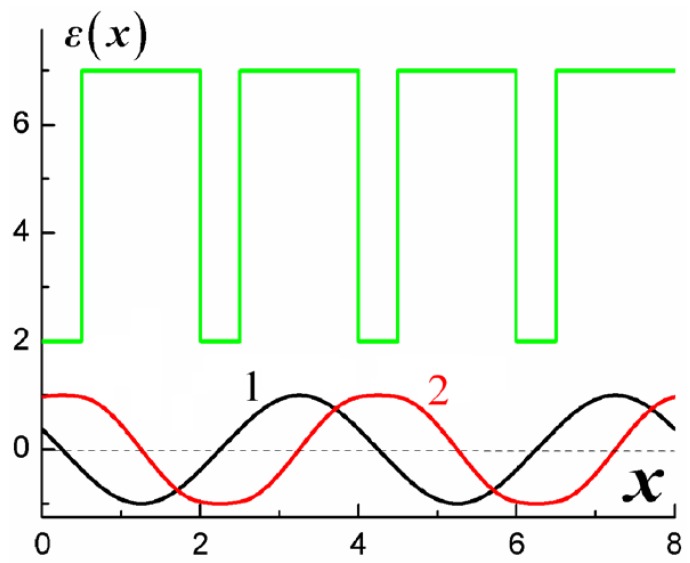
The results of computer simulation: the amplitude of the electric field distribution for a Bloch wave in a PC at frequencies near band edges: **1**—below a BG and **2**—above a BG. The curve at the top is the function ε(x).

The optical Borrmann effect was theoretically predicted and experimentally verified at the edges of a Brillouin BG [37,38,39,40,41,42,56]. For 1D PC it was shown [35] that at the lower edge of the BG the energy of a Bloch wave concentrates mainly in layers with higher permittivity, whereas at the upper edge of the BG the energy concentrates mainly in layers with lower permittivity (see Figure 13). This is the so-called direct Borrmann effect.

For a qualitative explanation of the direct Borrmann effect in the lowest pass band let us use the homogenization theory [42]. According to this theory, the effective permittivity can be defined as [57,58]

(9) $$\varepsilon_{eff}\langle EE^{*}\rangle =\langle \varepsilon EE^{*}\rangle$$

where the brackets 〈〉 denote averaging over the volume of an elementary cell and $n_{eff}$ is the effective refractive index. Although homogenization theory should work at low frequencies only, nevertheless, Equation (9) was shown [42] to describe the behavior of the real part of the wave number above the first BG rather well. The lower $k_{0}^{-}$ and upper $k_{0}^{+}$ frequency edges of the first band gap correspond to the same wave number $k_{Bl}=\pi /\Lambda$, where the subscripts “+” and “−” denote top and bottom edges of a BG, respectively. Therefore, normalizing the ﬁelds at different frequencies by the condition $\langle E_{-}E_{-}^{*}\rangle =\langle E_{+}E_{+}^{*}\rangle =1$, we can write the following equality: $k_{Bl}=k_{0}^{-}n_{-}=k_{0}^{+}n_{+}$, where $n_{\pm }=\sqrt{\langle \varepsilon E_{\pm }E_{\pm }^{*}\rangle }$. As a result, the inequality $k_{0}^{-}<k_{0}^{+}$ leads to the inequality for reflection indices: $n_{-}>n_{+}$ or

$$\langle \varepsilon E_{-}E_{-}^{*}\rangle >\langle \varepsilon E_{+}E_{+}^{*}\rangle$$

Taking into account that due to the normalization condition, ${\langle E_{\pm }E_{\pm }^{*}\rangle }_{l}=1-{\langle E_{\pm }E_{\pm }^{*}\rangle }_{h}$ (h and l denote higher- and lower-permittivity layers), and that

$$\langle \varepsilon E_{-}E_{-}^{*}\rangle >\varepsilon_{h}{\langle E_{-}E_{-}^{*}\rangle }_{h}+\varepsilon_{l}{\langle E_{-}E_{-}^{*}\rangle }_{l},\;\langle \varepsilon E_{+}E_{+}^{*}\rangle >\varepsilon_{h}{\langle E_{+}E_{+}^{*}\rangle }_{h}+\varepsilon_{l}{\langle E_{+}E_{+}^{*}\rangle }_{l},$$

we arrive at the following inequalities:

$$\varepsilon_{h}{\langle E_{-}E_{-}^{*}\rangle }_{h}>\varepsilon_{h}{\langle E_{+}E_{+}^{*}\rangle }_{h},\;\varepsilon_{l}{\langle E_{-}E_{-}^{*}\rangle }_{l}<\varepsilon_{l}{\langle E_{+}E_{+}^{*}\rangle }_{l}.$$

Therefore the field and the energy concentration in the high-permittivity layer at the bottom of the BG is greater than that at the top of the BG, whereas the ﬁeld and the energy concentration in the lower permittivity layer is greater at the top of the BG than that at the bottom of the BG, as shown in Figure 13.

In [40,42] it was shown that the Borrmann effect at the boundaries of a Brillouin BG of an isotropic PC can be inverted: under certain conditions the energy concentration in the high-permittivity layer can become smaller at the bottom of the BG than that at the top of the BG. However the inverse Borrmann effect was proved never to take place at the edges of the first BG [40,42].

Here we present a study of the Borrmann effect at the boundaries of degenerate BGs which was never studied before to the best of our knowledge. The orthogonality condition (8) is not applicable to the couple of Bloch modes at the two edges of a DBG, because in general $k_{Bl}\left(k_{0}^{-}\right)\neq k_{Bl}\left(k_{0}^{+}\right)$ (see, for example, Figure 8). Thus at first sight it is not clear whether the Borrmann effect occur at the edges of DBGs.

First, we consider an anisotropic DBG. A PC under study has two uniaxial layers in a period with dielectric tensors in the principal axes being

$${\hat{\varepsilon }}_{1}=\left(\begin{array}{ccc} 4.0 & 0 & 0\\ 0 & 1.0 & 0\\ 0 & 0 & 1.0 \end{array}\right),{\hat{\varepsilon }}_{2}=\left(\begin{array}{ccc} 8.0 & 0 & 0\\ 0 & 4.0 & 0\\ 0 & 0 & 4.0 \end{array}\right)$$

**Figure 14:**
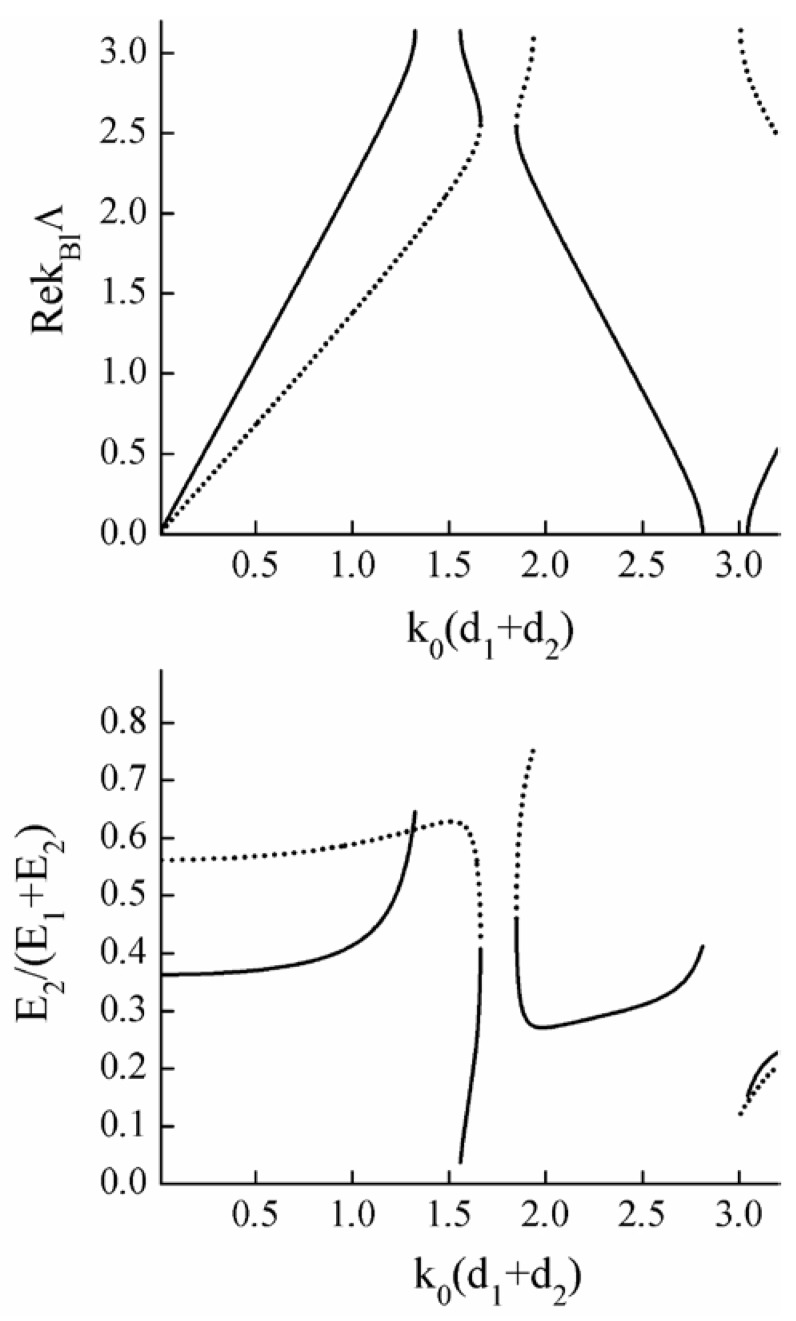
(Top figure) Dispersion curves of the PC with two anisotropic layers in a period. (Bottom figure) The relative part of the energy of Bloch waves concentrated in the second anisotropic layer of the PC unit cells. Solid and dotted curves correspond to two different Bloch modes. The degenerate BG forms around the frequency $k_{0}\left(d_{1}+d_{2}\right)\approx 1.76$. Only the parts of curves that lie in pass bands are shown.

The optical axes of the layers lie in the plane of the layers. The thickness of the first layer is $d_{1}$, the thickness of the second layer is $d_{2}=0.3d_{1}$. The angle between the optical axes of the layers is α=0.5 rad. Calculations of field distribution in the PC were carried out using the T-matrix method [29,53]. The results are presented in Figure 14, where the relative part of the energy of the Bloch waves concentrated in the PC layers of the second type is depicted. The degenerate BG forms around the frequency $k_{0}\left(d_{1}+d_{2}\right)\approx 1.76$.

Also, we consider a gyrotropic DBG. A PC under study has two layers in a period. One of them is an anisotropic uniaxial layer with dielectric tensors along the principal axes

$${\hat{\varepsilon }}_{a}=\left(\begin{array}{ccc} 3.5 & 0 & 0\\ 0 & 1.5 & 0\\ 0 & 0 & 1.5 \end{array}\right)$$

The other is gyrotropic with dielectric permittivity $\varepsilon_{g}=4$. The dielectric tensor for the gyrotropic layer is ${\hat{\varepsilon }}_{g}=\left(\begin{array}{ccc} \varepsilon_{g} & ig & 0\\ -ig & \varepsilon_{g} & 0\\ 0 & 0 & \varepsilon_{g} \end{array}\right)$.

**Figure 15:**
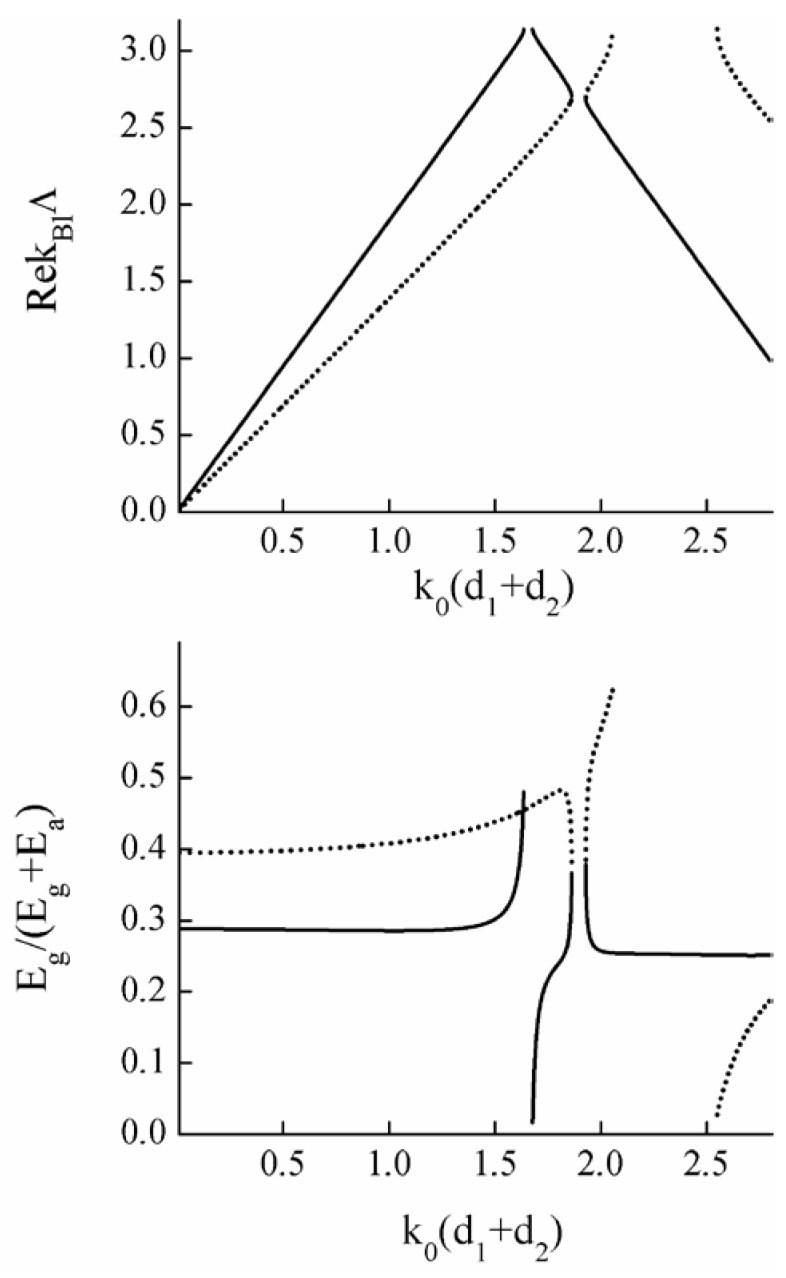
(Top figure) Dispersion curves of the PC with one anisotropic and one gyrotropic layer in a period. (Bottom figure) The relative part of the energy of the Bloch waves concentrated in the gyrotropic PC layers. Solid and dotted curves correspond to two different Bloch modes. The degenerate BG forms around the frequency $k_{0}\left(d_{1}+d_{2}\right)\approx 1.89$. Only the parts of curves that lie in pass bands are shown.

The thickness of the anisotropic layer is $d_{1}$, the thickness of the gyrotropic layer is $d_{2}=0.2d_{1}$. An external magnetic field is applied normal to the layers and g=0.5. Calculations of the field distribution in the PC were carried out using the T-matrix method. The results are presented in Figure 15, where the relative part of the energy of Bloch waves concentrated in the gyrotropic layers is depicted. The degenerate BG forms around the frequency $k_{0}\left(d_{1}+d_{2}\right)\approx 1.89$.

Figure 14 and Figure 15 show that the energy-distribution of each Bloch-mode in the PC elementary-cells change similarly when approaching either from the bottom or the top edges of the DBGs. Thus we conclude that in the general case there is no Borrmann effect at the boundaries of DBGs for 1D photonic crystals.

## 4. Formation of the Tamm States Based on Degenerate Band Gap

Due to the existing analogy between PCs and crystalline solids, many phenomena, well-known in solid state physics, are now observed in photonic crystals (PC). In particular, the surface states of electrons predicted by I. Tamm in 1934 [59] have been intensively investigated both at optical and microwave frequencies in PCs [5,6,7,8,9,10,11,12,13,14,15,16]. The optical Tamm state can form at the interface of two different PCs or between a PC and a medium with negative permittivity or permeability. The frequency $k_{0}=\omega /c$ at which the Tamm state occurs lies at the intersection of the band gaps (BGs) of the first and second PCs and is determined by the equality of the PC input admittances of the evanescent Bloch waves composing this Tamm state:

$$Y_{1}\left(k_{0}\right)=Y_{2}\left(k_{0}\right)$$

where the input admittance $Y\left(k_{0}\right)$ (which is the electrodynamic-analog of the logarithmic derivative of the psi-function) of a Bloch wave is equal to the ratio of the tangential components of the electric and magnetic fields $H_{t}/E_{t}$. Thus, this equation is equivalent to the usual Maxwell boundary conditions.

The Tamm state consists of two evanescent Bloch waves dying out away from the interface [5]. In this section we study the structure of the Tamm state, which appears in the degenerate BG.

**Figure 16:**
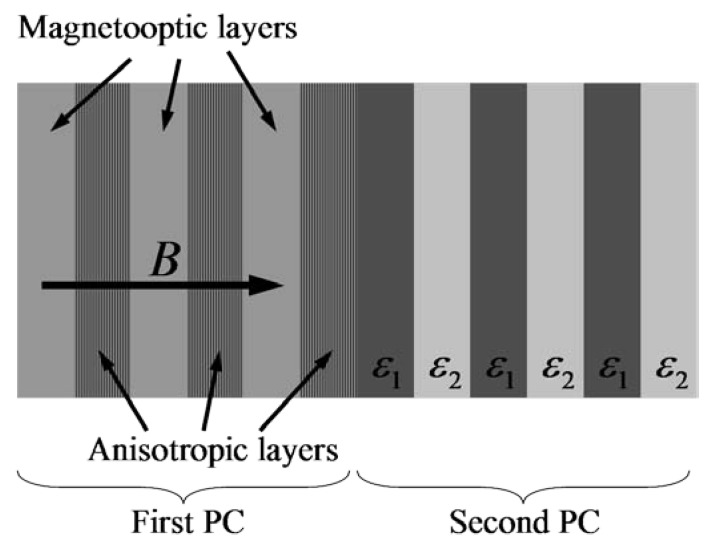
System under consideration. The unit cell of the first PC consists of a uniaxial crystal ($\varepsilon_{xx}=2.7$, $\varepsilon_{yy}=5.0$) and a magnetooptical layer ($\varepsilon_{diag}=3.0$, $\varepsilon_{off\_diag}=i\alpha =0.02i$ and $\varepsilon_{off\_diag}=0$ at zero magnetization). The unit cell of the second PC consists of two isotropic layers with permittivities $\varepsilon_{1}=3.1$ and $\varepsilon_{2}=3.4$. The thickness of each layer equals d.

Let us consider a Tamm state based on the DBG in a system consisting of an anisotropic magnetophotonic crystal (first PC in Figure 16) and a PC made up of isotropic components (second PC in Figure 16). The unit cell of the first PC consists of a uniaxial crystal and a magnetooptical layer and yields a degenerate BG. The unit cell of the second PC consists of two isotropic layers.

**Figure 17:**
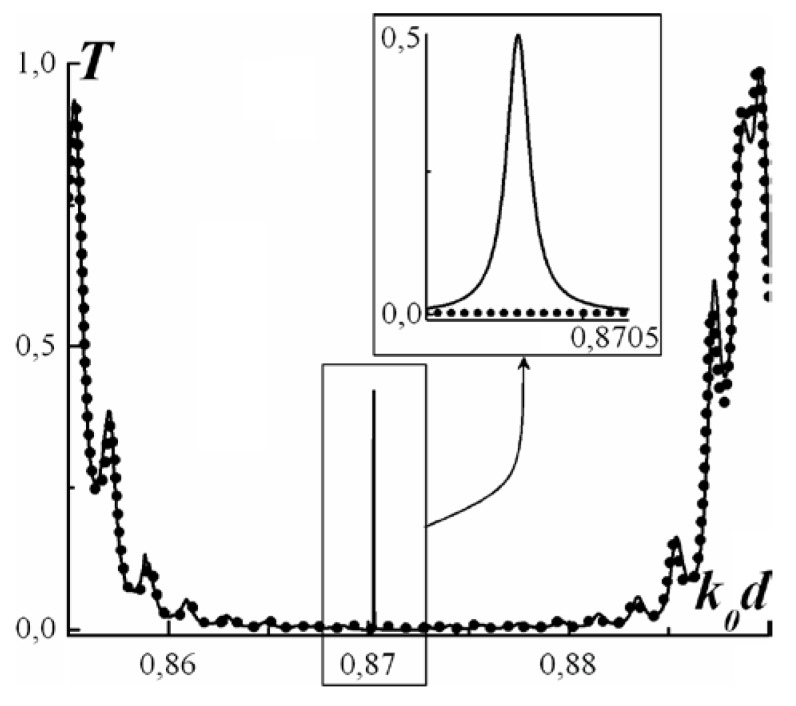
Propagation coefficient of the system under consideration. The dotted line corresponds to zero magnetization; the solid line corresponds to the magnetized case.

Let us consider the frequencies around the intersection of the dispersion curves of ordinary and extraordinary waves of a non-magnetized first PC. It is assumed that such frequencies correspond to the band gap of the second PC. At zero magnetization the transmittance is suppressed by the Bragg reflectance in the second PC (dotted line in Figure 17).

Application of a magnetic field results in the appearance of a degenerate band gap in the first PC and respectively leads to the formation of Tamm states at the boundary between the two PCs. Such a state reveals itself as a transparency peak in the transmittance spectrum (see Figure 17).

In the present case only one Tamm state appears as a result of magnetization, contrary to the case of a Tamm state at the boundary between an isotropic magnetooptical PC and a PC made up of isotropic components [5]. In the latter case, the Tamm state exists without magnetization and splits into two Tamm states upon magnetization (corresponding to right- and left-circularly polarized waves). The present Tamm state is not doubly degenerate with respect to polarization like the Tamm state at the boundary between two PCs made of isotropic layers. Such peculiarities of the present Tamm state are caused by the hybrid nature of the eigensolutions in a PC, based on anisotropic and magnetooptical materials.

## 5. Anisotropy of Admittance

The Bloch waves in a 1D PC made up of anisotropic materials with coincident directions of anisotropy axes are TE or TM polarized, having different but scalar impedance as in the case of a homogeneous medium. A non-zero angle between the axes or the presence of gyrotropy results in hybridization of the Bloch waves. These hybrid Bloch waves have neither linear nor circular (elliptical) polarization. As a consequence, the problem cannot be reduced to two scalar problems with scalar (yet possibly different) impedance values. In the scalar problem the boundary conditions reduce to equality of the impedance values on both sides of an interface. 

In the case of a primitive cell consisting of layers with anisotropic and gyrotropic permittivities, the Bloch wave consists of four waves differing in polarization and traveling directions in each layer. Waves with different polarizations are characterized by different scalar impedance values. The Bloch wave, as a consequence, is characterized by an impedance value tensor. If the second PC is made up of isotropic material we can construct a couple of Bloch waves having any type of polarization: linear, circular or elliptical. In any case there are two Bloch functions with complementary polarizations and the same impedance value. The boundary conditions still reduce to equality of the input impedance values. It is obvious that to satisfy such a condition we have to employ hybrid Bloch waves in order to obtain a symmetric tensor of impedance values.

Let us deduce the required equality following from the boundary conditions and serving as a dispersion relation for the surface state. As mentioned above, two complementary Bloch waves in the anisotropic MPC have different Bloch wave numbers: $k_{1}\left(k_{0}\right)=q_{1}\left(k_{0}\right)+iq_{2}\left(k_{0}\right)$, $k_{2}\left(k_{0}\right)=-q_{1}\left(k_{0}\right)+iq_{2}\left(k_{0}\right)$ and different periodic factors:

$${\vec{E}}_{\alpha }=\left(\begin{array}{c} f_{\alpha x}\left(z\right)\\ f_{\alpha y}\left(z\right) \end{array}\right)e^{ik_{1}z},\;{\vec{H}}_{\alpha }=\left(\begin{array}{c} Y_{\alpha y}f_{\alpha y}\left(z\right)\\ -Y_{\alpha x}f_{\alpha x}\left(z\right) \end{array}\right)e^{ik_{1}z}$$

where $Y_{\alpha \beta }\left(z\right)=n_{\alpha }+\frac{{f^{\prime }}_{\alpha \beta }}{f_{\alpha \beta }ik_{0}}$ is a tensor of the local impedance values, α=1,2—is a label of the Bloch wave and β=x,y—is a label of the component. To satisfy the boundary conditions we have to identify the fields in the adjoining layers belonging to the different PCs. Below, for definiteness, we consider the case where the layer with anisotropic permittivity adjoins the PC made up of isotropic materials. The hybrid Bloch waves in this layer may be presented as a sum of two ordinary and two extraordinary waves traveling in opposite directions:

$${\vec{E}}_{\alpha }=\left(\begin{array}{c} E_{\alpha x}\\ E_{\alpha y} \end{array}\right)=A_{\alpha }\left(\begin{array}{c} 1\\ 0 \end{array}\right)e^{ik_{o}z}+B_{\alpha }\left(\begin{array}{c} 1\\ 0 \end{array}\right)e^{-ik_{o}z}+C_{\alpha }\left(\begin{array}{c} 0\\ 1 \end{array}\right)e^{ik_{e}z}+D_{\alpha }\left(\begin{array}{c} 0\\ 1 \end{array}\right)e^{-ik_{e}z},\;$$

and

$$k_{0}{\vec{H}}_{\alpha }=k_{0}\left(\begin{array}{c} H_{\alpha x}\\ H_{\alpha y} \end{array}\right)=k_{o}A_{\alpha }\left(\begin{array}{c} 0\\ 1 \end{array}\right)e^{ik_{o}z}-k_{o}B_{\alpha }\left(\begin{array}{c} 0\\ 1 \end{array}\right)e^{-ik_{o}z}+k_{e}C_{\alpha }\left(\begin{array}{c} 1\\ 0 \end{array}\right)e^{ik_{e}z}-k_{e}D_{\alpha }\left(\begin{array}{c} 1\\ 0 \end{array}\right)e^{-ik_{e}z},\;$$

where α=1,2 is a label of the Bloch wave $A_{i},B_{i},C_{i},D_{i}$ are the phasors of the plane waves, which are eigensolutions of the Maxwell equations in the layer, $k_{o}$ and $k_{e}$ are the wave numbers of ordinary and extraordinary waves. It is easy to see that in this representation the admittance tensor is diagonal with $Y_{\alpha x}=Y_{o}=H_{x}/E_{y}$ and $Y_{\alpha y}=Y_{e}=-H_{y}/E_{x}$. In the isotropic medium the corresponding admittance values are identical: $Y_{x}=H_{x}/E_{y}=k/k_{0}=-H_{y}/E_{x}=Y_{y}=Y_{i}$. Hence we cannot simultaneously satisfy all boundary conditions confining ourselves to a single Bloch wave in the MPC. We have to consider a linear combination of two complementary Bloch waves in the anisotropic MPC:

$$\vec{E}=a\left(\begin{array}{c} f_{1x}\left(z\right)\\ f_{1y}\left(z\right) \end{array}\right)e^{ik_{1}z}+b\left(\begin{array}{c} f_{2x}\left(z\right)\\ f_{2y}\left(z\right) \end{array}\right)e^{ik_{2}z}\text{ and }\vec{H}=a\left(\begin{array}{c} Y_{1y}f_{1y}\left(z\right)\\ -Y_{1x}f_{1x}\left(z\right) \end{array}\right)e^{ik_{1}z}+b\left(\begin{array}{c} Y_{2y}f_{2y}\left(z\right)\\ -Y_{2x}f_{2x}\left(z\right) \end{array}\right)e^{ik_{2}z}$$

Taking into account that in the PC layer adjoining the MPC, the Bloch wave has a form

$$\vec{E}=\left(\begin{array}{c} E_{x}\\ E_{y} \end{array}\right)=\left(\begin{array}{c} cf\left(z\right)\\ df\left(z\right) \end{array}\right)e^{ikz}\text{ and }\vec{H}=\left(\begin{array}{c} H_{x}\\ H_{y} \end{array}\right)=\left(\begin{array}{c} d\left(n+\frac{f^{\prime }}{fik_{0}}\right)f\\ -c\left(n+\frac{f^{\prime }}{fik_{0}}\right)f \end{array}\right)e^{ikz}$$

the boundary conditions can be written down as

$$\left\{ \begin{array}{l} a\left(\begin{array}{c} f_{1x}\\ f_{1y} \end{array}\right)+b\left(\begin{array}{c} f_{2x}\\ f_{2y} \end{array}\right)=\left(\begin{array}{c} cf\\ df \end{array}\right)\\ a\left(\begin{array}{c} Y_{1y}f_{1y}\\ -Y_{1x}f_{1x} \end{array}\right)e^{ik_{1}z}+b\left(\begin{array}{c} Y_{2y}f_{2y}\\ -Y_{2x}f_{2x} \end{array}\right)=\left(\begin{array}{c} dYf\\ -cYf \end{array}\right) \end{array}\right.$$

This linear system (with respect to a,b,c,d) has a non-zero solution if

$$\left|\begin{array}{cc} \left(Y_{1y}-Y\right)f_{1y} & \left(Y_{2y}-Y\right)f_{2y}\\ \left(Y_{1x}-Y\right)f_{1x} & \left(Y_{2x}-Y\right)f_{2x} \end{array}\right|=0$$

Thus, we obtain the equation that determines the frequency of the Tamm state in the degenerate BG. 

We may conclude that at the interface between a MPC, with primitive cell made up of a layer with anisotropic permittivity and a layer with gyrotropic permittivity magnetophotonic crystal, and a PC, with primitive cell made up of layers with isotropic permittivity, there may appear a surface Tamm state. The frequency of this state lies in the degenerate BG of the first PC and in the Brillouin BG of the second one. Contrary to the quantum case and the case when both BGs are of the Brillouin type, this Tamm state consists of three evanescent Bloch waves. The necessity of the third Bloch wave is a consequence of the different bases in the PCs.

## Appendix

Here we solve Maxwell Equation (6) using perturbation theory with the assumptions that the periodic alterations $\Delta \varepsilon_{x}(z)$, $\Delta \varepsilon_{y}(z)$, Δg(z) in the functions $\varepsilon_{x}(z)$, $\varepsilon_{y}(z)$, g(z) from (5) are small compared to their average values $\varepsilon_{x0}=\varepsilon_{x}(z)-\Delta \varepsilon_{x}(z)$, $\varepsilon_{y0}=\varepsilon_{y}(z)-\Delta \varepsilon_{y}(z)$, $\varepsilon_{0}=g(z)-\Delta g(z)$, independent on z, and that α(z) is small compared to π. (The average value of function $\varepsilon_{x}(z)$ is its zero Fourier coefficient $\varepsilon_{x0}=\frac{1}{\Lambda }\int_{0}^{\Lambda }\varepsilon_{x}(z)dz$, where Λ is a PC period. Similarly, $\varepsilon_{y0}$, $\varepsilon_{0}$, $\alpha_{0}$ are calculated. It is assumed that $\alpha_{0}=0$). Then (6) is approximately equivalent to:

(A1) $$\begin{array}{l} \frac{d^{2}E_{x}}{dz^{2}}+k_{0}^{2}\varepsilon_{x0}E_{x}-ik_{0}^{2}\varepsilon_{0}E_{y}+k_{0}^{2}\Delta \varepsilon_{x}(z)E_{x}+k_{0}^{2}\left(\left(\varepsilon_{x0}-\varepsilon_{y0}\right)\alpha (z)-i\Delta \varepsilon (z)\right)E_{y}=0\\ \frac{d^{2}E_{y}}{dz^{2}}+k_{0}^{2}\varepsilon_{y0}E_{y}+ik_{0}^{2}\varepsilon_{0}E_{x}+k_{0}^{2}\Delta \varepsilon_{y}(z)E_{y}+k_{0}^{2}\left(\left(\varepsilon_{x0}-\varepsilon_{y0}\right)\alpha (z)+i\Delta \varepsilon (z)\right)E_{x}=0 \end{array}$$

These equations may be written in a more suitable form as follows. In a basis set of elliptically polarized waves $E_{r}=E_{x}-i\varphi E_{y}$ and $E_{l}=E_{y}-i\varphi E_{x}$ [22] (where $\varphi =\{{\left[{\left(\varepsilon_{x0}-\varepsilon_{y0}\right)}^{2}+4\varepsilon_{0}^{2}\right]}^{1/2}-(\varepsilon_{x0}-\varepsilon_{y0})\}/(2\varepsilon_{0})$) Equation (A1) read as:

(A2) $$\begin{array}{l} \frac{d^{2}E_{r}}{dz^{2}}+k_{1}^{2}E_{r}+k_{0}^{2}A_{r}(z)E_{r}+ik_{0}^{2}B(z)E_{l}+k_{0}^{2}\left(\varepsilon_{x0}-\varepsilon_{y0}\right)\alpha (z)E_{l}=0\\ \frac{d^{2}E_{l}}{dz^{2}}+k_{2}^{2}E_{l}+k_{0}^{2}A_{l}(z)E_{l}-ik_{0}^{2}B(z)E_{r}+k_{0}^{2}\left(\varepsilon_{x0}-\varepsilon_{y0}\right)\alpha (z)E_{r}=0 \end{array}$$

Here $A_{r}(z)=\frac{\Delta \varepsilon_{x}(z)+2\varphi \;\Delta \varepsilon (z)+\varphi^{2}\Delta \varepsilon_{y}(z)}{1+\varphi^{2}}$, $A_{l}(z)=\frac{\Delta \varepsilon_{y}(z)-2\varphi \;\Delta \varepsilon (z)+\varphi^{2}\Delta \varepsilon_{x}(z)}{1+\varphi^{2}}$, $B(z)=\frac{\left(\Delta \varepsilon_{x}(z)-\Delta \varepsilon_{y}(z))\varphi +\Delta \varepsilon (z)(\varphi^{2}-1\right)}{1+\varphi^{2}}$, $\frac{k_{1}^{2}}{k_{0}^{2}}=n_{1}^{2}=\frac{1}{2}\left(\varepsilon_{x0}+\varepsilon_{y0}+{\left[{\left(\varepsilon_{x0}-\varepsilon_{y0}\right)}^{2}+4\varepsilon_{0}^{2}\right]}^{1/2}\right)$, $\frac{k_{2}^{2}}{k_{0}^{2}}=n_{2}^{2}=\frac{1}{2}\left(\varepsilon_{x0}+\varepsilon_{y0}-{\left[{\left(\varepsilon_{x0}-\varepsilon_{y0}\right)}^{2}+4\varepsilon_{0}^{2}\right]}^{1/2}\right)$.

In Equation (A2) small perturbations are described by functions $A_{r}(z)$, $A_{l}(z)$, B(z), α(z), due to the smallness of $\Delta \varepsilon_{x}(z)$, $\Delta \varepsilon_{y}(z)$, Δg(z). Equation (A2) in contrast to Equations A1 are coupled only by perturbation terms. The coupling between Equation (A2) describes hybridization of differently polarized (with right or left elliptic polarization) components of Bloch waves. This coupling is the necessary condition for a DBG to form.

As the medium is periodic, a general solution can be expanded as a sum of Bloch waves:

(A3) $$E=\left(\begin{array}{c} E_{r}(z)\\ E_{l}(z) \end{array}\right)=\sum_{j=-\infty }^{+\infty }\left(\begin{array}{c} R_{j}\\ L_{j} \end{array}\right)\exp(i(k_{Bl}+j\varepsilon )z)$$

where ε=2π/Λ is a reciprocal lattice parameter. Expanding the perturbation functions into Fourier series $A_{r}(z)=\sum_{j=-\infty }^{+\infty }A_{r,j}\exp(ij\varepsilon z)$, $A_{l}(z)=\sum_{j=-\infty }^{+\infty }A_{l,j}\exp(ij\varepsilon z)$, $B(z)=\sum_{j=-\infty }^{+\infty }B_{j}\exp(ij\varepsilon z)$, $\alpha (z)=\sum_{j=-\infty }^{+\infty }\alpha_{j}\exp(ij\varepsilon z)$, then substituting along with (A3) into (A2), collecting similar terms and setting the coefficients before each Fourier harmonic equal to zero we come to an infinite system of coupled linear algebraic equations:

(A4) $$\begin{array}{l} -{\left(k_{Bl}+j\varepsilon \right)}^{2}R_{j}+k_{1}^{2}R_{j}+k_{0}^{2}\sum_{j\prime =-\infty }^{+\infty }A_{r,j-j\prime }R_{j\prime }+ik_{0}^{2}\sum_{j\prime =-\infty }^{+\infty }B_{j-j\prime }L_{j\prime }+k_{0}^{2}\left(\varepsilon_{x0}-\varepsilon_{y0}\right)\sum_{j\prime =-\infty }^{+\infty }\alpha_{j-j\prime }L_{j\prime }=0\\ -{\left(k_{Bl}+j\varepsilon \right)}^{2}L_{j}+k_{2}^{2}L_{j}+k_{0}^{2}\sum_{j\prime =-\infty }^{+\infty }A_{l,j-j\prime }L_{j\prime }-ik_{0}^{2}\sum_{j\prime =-\infty }^{+\infty }B_{j-j\prime }R_{j\prime }+k_{0}^{2}\left(\varepsilon_{x0}-\varepsilon_{y0}\right)\sum_{j\prime =-\infty }^{+\infty }\alpha_{j-j\prime }R_{j\prime }=0 \end{array}$$

The simultaneous fulfillment of two conditions:

(A5) $$k_{1}^{2}-k_{Bl}^{2}\approx 0\text{ and }k_{2}^{2}-{\left(k_{Bl}-\varepsilon \right)}^{2}\approx 0$$

(A6) $$\text{or }k_{2}^{2}-k_{Bl}^{2}\approx 0\text{ and }k_{1}^{2}-{\left(k_{Bl}-\varepsilon \right)}^{2}\approx 0$$

at a frequency $k_{0}$ was shown [27] to correspond to DBG formation. The Equations (A5) (or (A6)) together are analogous to Equation (A2), thus being the conditions for a Bragg reflection in the case of a low contrast PC. We can recast Equation (A4) as:

$$R_{j}=\frac{k_{0}^{2}\sum_{\begin{array}{l} j\prime =-\infty \\ j\prime \neq j \end{array}}^{+\infty }A_{r,j-j\prime }R_{j\prime }+ik_{0}^{2}\sum_{\begin{array}{l} j\prime =-\infty \\ j\prime \neq j \end{array}}^{+\infty }B_{j-j\prime }L_{j\prime }+k_{0}^{2}\left(\varepsilon_{x0}-\varepsilon_{y0}\right)\sum_{\begin{array}{l} j\prime =-\infty \\ j\prime \neq j \end{array}}^{+\infty }\alpha_{j-j\prime }L_{j\prime }}{{\left(k_{Bl}+j\varepsilon \right)}^{2}-k_{1}^{2}},$$

$$L_{j}=\frac{k_{0}^{2}\sum_{\begin{array}{l} j\prime =-\infty \\ j\prime \neq j \end{array}}^{+\infty }A_{l,j-j\prime }L_{j\prime }-ik_{0}^{2}\sum_{\begin{array}{l} j\prime =-\infty \\ j\prime \neq j \end{array}}^{+\infty }B_{j-j\prime }R_{j\prime }+k_{0}^{2}\left(\varepsilon_{x0}-\varepsilon_{y0}\right)\sum_{\begin{array}{l} j\prime =-\infty \\ j\prime \neq j \end{array}}^{+\infty }\alpha_{j-j\prime }R_{j\prime }}{{\left(k_{Bl}+j\varepsilon \right)}^{2}-k_{2}^{2}}.$$

Then, under the simultaneous fulfillment of conditions (A5) the denominators for j=−1 and j=0 simultaneously tend to zero for $R_{0}$ and $L_{-1}$. Thus we can [27,29] confine ourselves to consideration of only two equations for the harmonics $R_{0}$ and $L_{-1}$:

(A7) $$\begin{array}{l} (k_{1}^{2}-k_{Bl}^{2})R_{0}+ik_{0}^{2}B_{1}L_{-1}+\left(\varepsilon_{x0}-\varepsilon_{y0}\right)k_{0}^{2}\alpha_{1}L_{-1}=0\\ (k_{2}^{2}-{\left(k_{Bl}-\varepsilon \right)}^{2})L_{-1}-ik_{0}^{2}B_{-1}R_{0}+\left(\varepsilon_{x0}-\varepsilon_{y0}\right)k_{0}^{2}\alpha_{-1}R_{0}=0 \end{array}$$

Equating the determinant of the system (A7) to zero we derive the dispersion equation for Bloch waves near a DBG:

(A8) $$(k_{1}^{2}-k_{Bl}^{2})(k_{2}^{2}-{\left(k_{Bl}-\varepsilon \right)}^{2})-k_{0}^{4}{\left|\left(\varepsilon_{x0}-\varepsilon_{y0}\right)\alpha_{1}+iB_{1}\right|}^{2}=0$$

here we present inferences from Equation (A8) (for a detailed analysis see Reference [27]). The center of the DBG is at a frequency $k_{0}^{*}=\varepsilon /(n_{1}+n_{2})$ (it corresponds to a simultaneous fulfillment of Equation (A5)). If the second term in Equation A8 is unequal to zero then the Bloch wave number has an imaginary part at this frequency. We can find frequencies of the DBG boundaries as frequencies at which the imaginary part of the Bloch wave number vanishes, and therefore find the BG width. This is easy under the assumption of smallness of the second term in Equation (A8).

The combined DBG width is then determined by

(A9) $$\Delta k_{0}^{\varepsilon A}=\frac{2k_{0}^{*}\left|\left(\varepsilon_{x0}-\varepsilon_{y0}\right)\alpha_{1}+iB_{1}\right|}{\left(n_{1}+n_{2}\right){\left(n_{1}n_{2}\right)}^{1/2}}$$

Now we are ready to evaluate the contribution of each BG formation mechanism to the width of the combined DBG and to answer the question whether one mechanism can compensate the other.

First consider the case of a PC with two layers in a period. So the functions $\varepsilon_{x}(z)$, $\varepsilon_{y}(z)$, g(z), α(z) are piecewise constant functions of z. They are constant inside each layer of a PC elementary cell and change weakly from layer to layer. In the case of a bilayer PC the coordinates z may be chosen in a way that all the functions $\varepsilon_{x}(z)$, $\varepsilon_{y}(z)$, g(z), α(z) (and therefore the function B(z)) become even. Then coefficients $B_{1}$ and $\alpha_{1}$ are real and the formula (A9) transforms to

(A10) $$\Delta k_{0}^{\varepsilon A}=\frac{2k_{0}^{*}{\left[{\left(\varepsilon_{x0}-\varepsilon_{y0}\right)}^{2}\alpha_{1}^{2}+B_{1}^{2}\right]}^{1/2}}{\left(n_{1}+n_{2}\right){\left(n_{1}n_{2}\right)}^{1/2}}$$

If only the first mechanism of hybridization is present, *i.e.*, when Δα(z)≠0, but g(z)≡0, we derive the width of the anisotropic DBG Equation (A10):

$$\Delta k_{0}^{A}=\frac{2\left|\varepsilon_{x0}^{1/2}-\varepsilon_{y0}^{1/2}\right|\left|\alpha_{1}\right|\varepsilon }{\left(\varepsilon_{x0}^{1/2}+\varepsilon_{y0}^{1/2}\right)\varepsilon_{x0}^{1/4}\varepsilon_{y0}^{1/4}}$$

If $\varepsilon_{0}<<\left|\varepsilon_{x0}-\varepsilon_{y0}\right|$ (which is true in almost all cases due to the smallness of ε in real dielectrics), then $n_{1}\approx \varepsilon_{0x}^{1/2}$ and $n_{2}\approx \varepsilon_{0y}^{1/2}$, so

(A11) $$\Delta k_{0}^{A}\approx \frac{2k_{0}^{*}\left|\left(\varepsilon_{x0}-\varepsilon_{y0}\right)\alpha_{1}\right|}{\left(n_{1}+n_{2}\right){\left(n_{1}n_{2}\right)}^{1/2}}$$

If only the second mechanism is present, *i.e.*, when Δα(z)≡0, we derive the gyrotropic DBG width from Equation (A10):

(A12) $$\Delta k_{0}^{\varepsilon }=\frac{2k_{0}^{*}\left|B_{1}\right|}{\left(n_{1}+n_{2}\right){\left(n_{1}n_{2}\right)}^{1/2}}$$

Hence from Equations (A10, A11, A12) we get:

(A13) $$\Delta k_{0}^{\text{GA}}={\left[{\left(\Delta k_{0}^{\text{A}}\right)}^{2}+{\left(\Delta k_{0}^{\text{G}}\right)}^{2}\right]}^{\text{1/2}}$$

If there are three or more layers in a PC unit cell, a linkage between DBGs of different types (anisotropic and gyrotropic) could be more complicated. It follows for Equation (A9). Consider a PC with four layers of the same thickness in a unit cell. Take the functions $\varepsilon_{x}(z)$, $\varepsilon_{y}(z)$, g(z), α(z) in a way that in the first and the second layers $\alpha (z)=\alpha^{first}$ and in the third and the fourth layers $\alpha (z)=\alpha^{third}$, and all the functions, $\varepsilon_{x}(z)$, $\varepsilon_{y}(z)$, $g_{y}(z)$ are equal, respectively, to $\varepsilon_{x}^{first}$, $\varepsilon_{y}^{first}$, $g^{first}$ in the first and the fourth layers and to $\varepsilon_{x}^{third}$, $\varepsilon_{y}^{third}$, $g^{first}$ in the second and the third layers. Thus, if we set the origin of the coordinate z to be in the center of one of the unit cells then the functions $\varepsilon_{x}(z)$, $\varepsilon_{y}(z)$, g(z) (and therefore B(z)) are even and the function α(z) is odd. So *B*_1_ is real and $\alpha_{1}$ is pure imaginary, and the numerator in Equation (A9) can be zero. It means closing of the DBG. It is worth mentioning that the formula for $\Delta k_{0}^{\text{GA}}$ depends on the choice of Bragg conditions. If we take the second pair of Bragg c Equation (A6) instead of Equation (A5) then Equation (A9) is replaced by

(A14) $$\Delta k_{0}^{\varepsilon A}=\frac{2k_{0}^{*}\left|\left(\varepsilon_{x0}-\varepsilon_{y0}\right)\alpha_{1}-iB_{1}\right|}{\left(n_{1}+n_{2}\right){\left(n_{1}n_{2}\right)}^{1/2}}$$

The choice between the two pairs of Bragg Equations ((A5) or (A6)) does not affect the width of the DBG in the case of a bilayer PC, but is important if a unit cell of a PC is more complex. This choice determines the signs of the real parts of Bloch wave numbers in the reduced Brillouin zone at the frequencies of a DBG. So if Equation (A5) are met and $\Delta k_{0}^{\varepsilon A}=0$ from Equation (A9) then no DBG form for e.g., RekBl>0 (see the Figure 12). But for this PC (A14) gives for the width of the DBG at RekBl<0.
